# Carboplatin +/− Topotecan Ophthalmic Artery Chemosurgery for Intraocular Retinoblastoma

**DOI:** 10.1371/journal.pone.0072441

**Published:** 2013-08-21

**Authors:** Jasmine H. Francis, Y. Pierre Gobin, Ira J. Dunkel, Brian P. Marr, Scott E. Brodie, Gowtham Jonna, David H. Abramson

**Affiliations:** 1 Ophthalmic Oncology Service, Memorial Sloan-Kettering Cancer Center, New York, New York, United States of America; 2 Service of Interventional Neuroradiology, Departments of Neurosurgery Neurology and Radiology, Weill Cornell Medical College of New York Presbyterian Hospital, New York, New York, United States of America; 3 Department of Pediatrics, Memorial Sloan-Kettering Cancer Center, New York, New York, United States of America; 4 Department of Pediatrics, Weill Cornell Medical College, New York, New York, United States of America; 5 Department of Ophthalmology, Weill Cornell Medical College, New York, New York, United States of America; 6 Department of Ophthalmology, Mount Sinai School of Medicine, New York, New York, United States of America; Massachusetts Eye & Ear Infirmary, Harvard Medical School, United States of America

## Abstract

**Purpose:**

Carboplatin administered systemically or periocularly can result in dramatic and prompt regression of retinoblastoma. However, both routes are rarely curative alone and have undesirable side effects. We aimed to assess the efficacy and toxicity of carboplatin +/− topotecan delivered by ophthalmic artery chemosurgery whereby chemotherapy is infused into the eye via the ophthalmic artery.

**Methods:**

This retrospective, IRB-approved study investigated retinoblastoma patients whom received carboplatin +/− topotecan ophthalmic artery chemosurgery. Patient survival, ocular survival, hematologic toxicity, ocular toxicity, second cancer development and electroretinogram response were all evaluated.

**Results:**

57 carboplatin +/− topotecan infusions (of 111 total) were performed in 31 eyes of 24 patients. The remaining infusions were melphalan-containing. All patients were alive and no patient developed a second malignancy at a median follow up of 25 months. The Kaplan-Meier estimate of ocular survival at two years was 89.9% (95% confidence interval [CI], 82.1–97.9%) for all eyes. Grade 3 or 4 neutropenia developed in two patients and one patient developed metastatic disease. By univariate analysis, neither increasing maximum carboplatin/topotecan dose nor cumulative carboplatin/topotecan dose was associated with statistically significant reduction in the electroretinogram responses.

**Conclusion:**

Carboplatin +/− topotecan infusions are effective for ophthalmic artery chemosurgery in retinoblastoma: they demonstrate low hematologic and ocular toxicity and no statistically significant influence on electroretinogram responses, and used in conjunction with melphalan-containing OAC, demonstrate excellent patient survival and satisfactory ocular survival.

## Introduction

More than 25 years ago, oncologists worldwide began searching for alternatives to external beam irradiation as primary treatment for intraocular retinoblastoma, in part to avoid the associated risk of second cancers [1]–[4]. Although systemic chemotherapy had been demonstrated to cause dramatic local responses in 1953 [5], it was repeatedly used and then abandoned because of significant side effects (including death). That changed when Doz and colleagues introduced the use of systemic carboplatin in combination with other drugs [6], [7]. This approach was quickly adopted in the U.S. [8] and by 2001 more than 100 peer-reviewed publications had confirmed that a carboplatin based chemotherapy regimen was effective (but not curative) in causing regression of intraocular tumors; and when combined with focal treatments (laser, cryotherapy and/or brachytherapy) could be effective in curing many intraocular tumors. Not only could carboplatin combined with other drugs cause tumor regression, but single agent carboplatin-administered intravenously [8], [9] or by peri-ocular injection also caused regression [10].

At the same time a novel and very different approach was being pursued in a few centers worldwide. Since retinoblastoma appeared to be so chemosensitive and in higher income countries presented as localized ocular disease in >95% of cases, attempts to deliver chemotherapy to the eye alone were explored. All blood to the retina comes from one blood vessel (the ophthalmic artery – almost always a branch of the internal carotid artery) so clinicians began exploring ways of selectively delivering chemotherapy to that artery alone. Reese began this in the 1950’s by injecting Triethelene melanamine into the carotid artery [11] and then “selective ophthalmic artery chemotherapy” was developed by Japanese investigators [12]. Based on an in vitro assay they determined that melphalan was potentially the most effective drug to use [13]; and multiple papers from Japan and their 25-year results [14] confirmed its efficacy clinically.

In 2006 we introduced the first successful intraarterial delivery of chemotherapy for retinoblastoma by entering the ophthalmic artery itself (stopping at the ostium) [15]. Due to the success the Japanese demonstrated with melphalan, we built on their work and, like them, began using melphalan. Nearly seven years later our 20 publications summarizing success in over 700 infusions and the multiple papers from other centers worldwide, which adopted our technique, drug and dosage have confirmed the potency of melphalan for this approach [16].

We had been impressed with the efficacy of intravenous carboplatin for retinoblastoma, and in an effort to minimize systemic effects and increase its local efficacy, we began using carboplatin intraarterially (starting in 2007) in combination with other drugs and then as a single agent. In our 2008 report we mentioned two cases that had received intraarterial carboplatin [15] and ten other papers from our institution have mentioned its use in our center [16]–[26]. Likewise, we chose to use topotecan intraarterially following promising evidence of its activity against retinoblastoma. This has been demonstrated in cells in culture [27], in a rodent model [28], and in children with both intraocular and refractory/relapsed metastatic disease [29]. As a topoisomerase inhibitor, topotecan results in DNA strand breakage and renders cells more susceptible to other chemotherapeutic agents. As a result, it is best used in combination, and we therefore use it only in conjunction with melphalan or carboplatin. We have now had experience with more than 70 infusions of carboplatin +/− topotecan and longer follow-up (as long as five years and nine months) and have now retrospectively collected our results for this report.

## Methods

Ethics Statement: Written informed consent was obtained from the parents, caretakers or guardians on behalf of all children and placed into the patient record. Parents/caretakers/guardians gave consent for this established, published treatment for an off-label use of chemotherapy. The Memorial Sloan-Kettering Institutional Review Board provided an exemption for the retrospective review of these patients. Patients would have received these treatments regardless of their inclusion in this study. All authors, who represented the team of physicians caring for these patients, collectively made the treatment decision.

Inclusion criteria consisted of eyes that had received carboplatin alone or in combination with topotecan. Eyes treated with all three drugs in a single OAC cycle have been reported separately and were excluded from this study [24]. One eye which received carboplatin only in the first OAC cycle followed by methotrexate only in the second OAC was included. A total of 111 OAC infusions were given to 31 eyes, 57 of which were carboplatin +/− topotecan-containing OAC infusions and 56 of which were infusions of melphalan +/− topotecan. This retrospective, single institution, study included all eyes of retinoblastoma patients meeting the inclusion criteria treated at Memorial Sloan Kettering and New York Presbyterian Hospital Weill-Cornell from May 30 2006 to May 30 2012. Patient data included age, sex, laterality, weight, treatment status (naïve vs prior treatment involving systemic chemotherapy or radiation), age at first OAC, follow-up time. Tumor data included Reese-Ellsworth (RE) classification, International Classification (IC), focal treatment and response to treatment.

Eyes were examined under anesthesia at three to four week intervals. Assessment consisted of visual assessment, motility and pupillary responses, indirect ophthalmoscopy, fundus photography with RetCam (Massie Industries, Dublin, CA), ophthalmic ultrasonography (OTI ophthalmic technologies, Inc. Toronto, Canada) and electroretinography (Espion ColorBurst, Diagnosys LLC, Lowell, MA). OAC was performed every three or four weeks in a manner that has previously been described in detail [17]. Response to carboplatin was assessed in the naïve eyes that received carboplatin with or without topotecan therapy during their initial infusion. This was evaluated by measuring the change in largest basal diameter of the tumor following this initial carboplatin +/− topotecan-containing OAC.

Baseline electroretinogram measurements were compared to recordings obtained at both three months and one year after completion of OAC therapy and were available for 29 eyes (one eye was enucleated prior to three months following OAC completion and two other eyes did not have baseline ERGs). Univariate regression analysis of ERG change with maximum and cumulative carboplatin doses was performed. Student’s t-test was used to analyze the change in ERG response before and after each cycle of carboplatin-only infusions (19 of 22 infusions evaluable) and carboplatin with topotecan infusions (31 of 35 infusions evaluable). Reported here are the response amplitudes to 30-Hz photopic flicker stimulation, which are representative of the full ERG protocol. As previously described [18], ERG amplitudes were classified according to the following scale: 0: undetectable; 0.1–25 µV: poor; 25.1–50 µV: fair; 50.1–75 µV: good; 75.1–100 µV: very good; >100 µV: excellent.

The standard Common Terminology Criteria for Adverse Events (CTCAE) v4.0 was used to grade hematologic toxicity. Of the three drugs employed at the current doses used for OAC, melphalan has the highest systemic dose toxicity profile. Therefore, to better isolate the contribution of carboplatin on systemic toxicity, this report restricts its assessment of systemic toxicity to those cases in which the unilateral or bilateral infusions consisted of carboplatin alone or carboplatin with topotecan. Cycles were considered evaluable for hematopoietic toxicity if a complete blood count was available between seven and 14 days post-infusion or, if a grade 3 or 4 toxicity was noted, outside of that time interval.

Statistical analysis was performed using the paired Student’s T-test and univariate regression analysis with a significance level of p-value <0.05. Kaplan-Meier survival analysis with a log-rank test was used to estimate ocular survival using Graphpad Prism (www.graphpad.com). An ocular event was defined as enucleation or external beam radiation.

## Results

**Figure 1:**
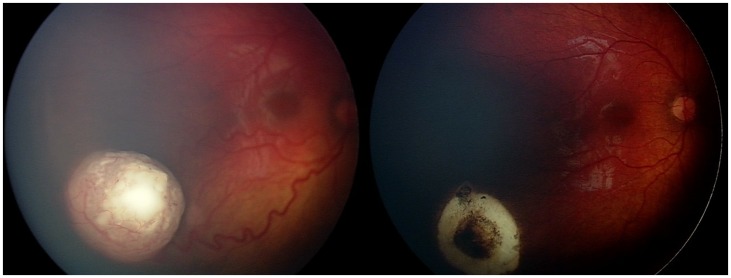
Response to carboplatin +/− topotecan-containing ophthalmic artery chemosurgery (OAC). Representative case. Left: Reese-Ellsworth Group VB (IC D) left eye prior to treatment Right: Same eye following three OAC cycles consisting of carboplatin and topotecan. Note dramatic response of tumors following carboplatin/topotecan therapy.

A total of 57 carboplatin +/− topotecan-containing OAC infusions were performed in 31 eyes of 24 retinoblastoma patients and their characteristics are presented in Table 1. Details pertaining to the carboplatin +/− topotecan-containing OAC treatments are depicted in Table 2. The mean age at first OAC was 15.4 months and the median was 10.5 months, with a range of three to 65 months. In six naïve eyes that received an initial carboplatin +/− topotecan-containing OAC treatment, the tumors decreased by a mean largest basal diameter of 29% following that first infusion. A representative example of retinoblastoma response to carboplatin based OAC is shown in figure 1.

**Table 1:** Carboplatin +/− topotecan-containing ophthalmic artery chemosurgery: Characteristics of patients, eyes and their infusions.

Patient Characteristics	24 patients, N (%)
Gender	
Male	12 (50)
Female	12 (50)
Disease Laterality	
Unilateral	7 (29)
Bilateral	17 (71)
Eye characteristics	31 eyes, N (%)
Prior Treatment	
Yes	14 (45)
No	17 (55)
Reese-Ellsworth Classification	
I-III	10 (32)
IV	3 (10)
Va	4 (13)
Vb	14 (45)
International Classification	
B	7 (23)
C	8 (26)
D	12 (39)
E	4 (13)
Infusion characteristics	57 infusions, N (%)
Drug Regimen	
Carboplatin alone	22 (39)
Carboplatin and Topotecan	35 (61)

**Table 2:** Characteristics of carboplatin +/− topotecan-containing infusions (percentage of carboplatin infusions, and maximum and cumulative carboplatin/topotecan doses) and subsequent focal treatment in eyes receiving carboplatin +/− topotecan-containing ophthalmic artery chemosurgery.

Pt/Eye	% COAC	Max C (mg)	Cum C (mg)	Max T (mg)	Cum T (mg)	Focal tx
1 R	50	50	95	0	0	2TTT/1Cr
2 L	33.3	30	60	0.4	2.1	plaque
3 L	20	30	30	0.4	1.6	TTT/plaque
4 L	25	30	30	0.3	1.2	none
5 L	33	30	30	0	0	none
5 R	25	30	30	0.4	1.4	5TTT
6 L	66.6	30	60	0.5	1.0	1TTT
7 R	66.6	30	60	0.5	0.5	3TTT
8 L	66.6	30	60	0.3	0.6	3TTT/1Cr
9 L	33.3	25	25	0	0	3TTT
10 L	66.6	30	60	0	0	2TTT
10 R	33.3	30	30	0.3	0.3	none
11 R	50	30	30	0.3	0.3	4TTT
11 L	25	25	25	0.3	1.2	3TTT
12 L	40	25	50	0.3	1.5	3TTT/1Cr
13 L	33.3	25	25	0.3	0.3	1TTT
14 R	66.6	25	50	0.3	0.6	1TTT
15 L	50	30	60	0.4	1.5	none
16 R	25	40	40	0.3	1.2	2TTT
16 L	100	40	100	0	0	4TTT
17 R	33.3	30	30	0.4	0.4	3TTT
18 L	83.3	40	150	0.5	1.0	2TTT/1Cr
19 R	100	30	30	0.0	0.0	3TTT/3Cr
20 R	100	30	55	0.3	0.3	2TTT
20 L	50	30	30	0.3	0.6	3TTT
21 L	80	30	120	1.0	3.0	5TTT
22 R	60	50	130	0.5	2.0	5TTT
22 L	33	40	40	0.5	1.0	5TTT
23 R	66	40	80	0.5	1.5	none
23 L	50	40	40	0.5	1.0	none
24 R	60	50	120	2.0	4.5	1TTT

pt =  patient, OAC =  ophthalmic artery chemosurgery, %COAC =  percentage of carboplatin-based OAC of all OAC infusions, Max C =  maximum carboplatin dose, Cum C =  cumulative carboplatin dose over all OAC infusions, Max T =  maximum topotecan dose, Cum T =  cumulative topotecann dose over all OAC infusions, focal tx =  treatment since beginning OAC, TTT =  transpupillary thermotherapy, Cr =  cryotherapy.

### Patient Survival

All children are alive and none developed second cancers. As previously reported, one patient developed metastatic disease, but is currently free of disease following intensive therapy [17]. Following the OAC procedure, there were no strokes, seizures or hospitalizations.

### Ocular Survival

**Figure 2:**
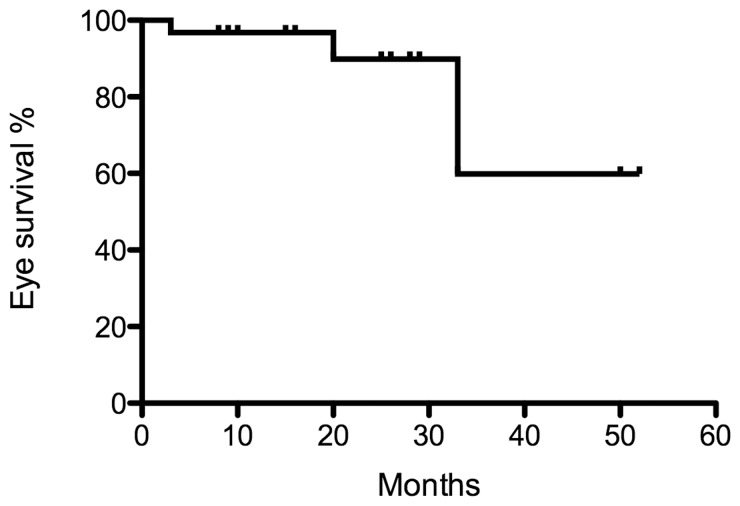
Kaplan-Meier ocular survival curve of all eyes.

The Kaplan-Meier ocular survival curve is shown in figure 2. The Kaplan-Meier estimate of ocular survival at two years was 89.9% (95% confidence interval [CI], 82.1–97.9%) for all eyes.

Three eyes were enucleated, none attributable to complications from the OAC procedure. These three eyes demonstrated progressive disease one, five and six months following the last OAC.

### Electroretinogram

**Figure 3:**
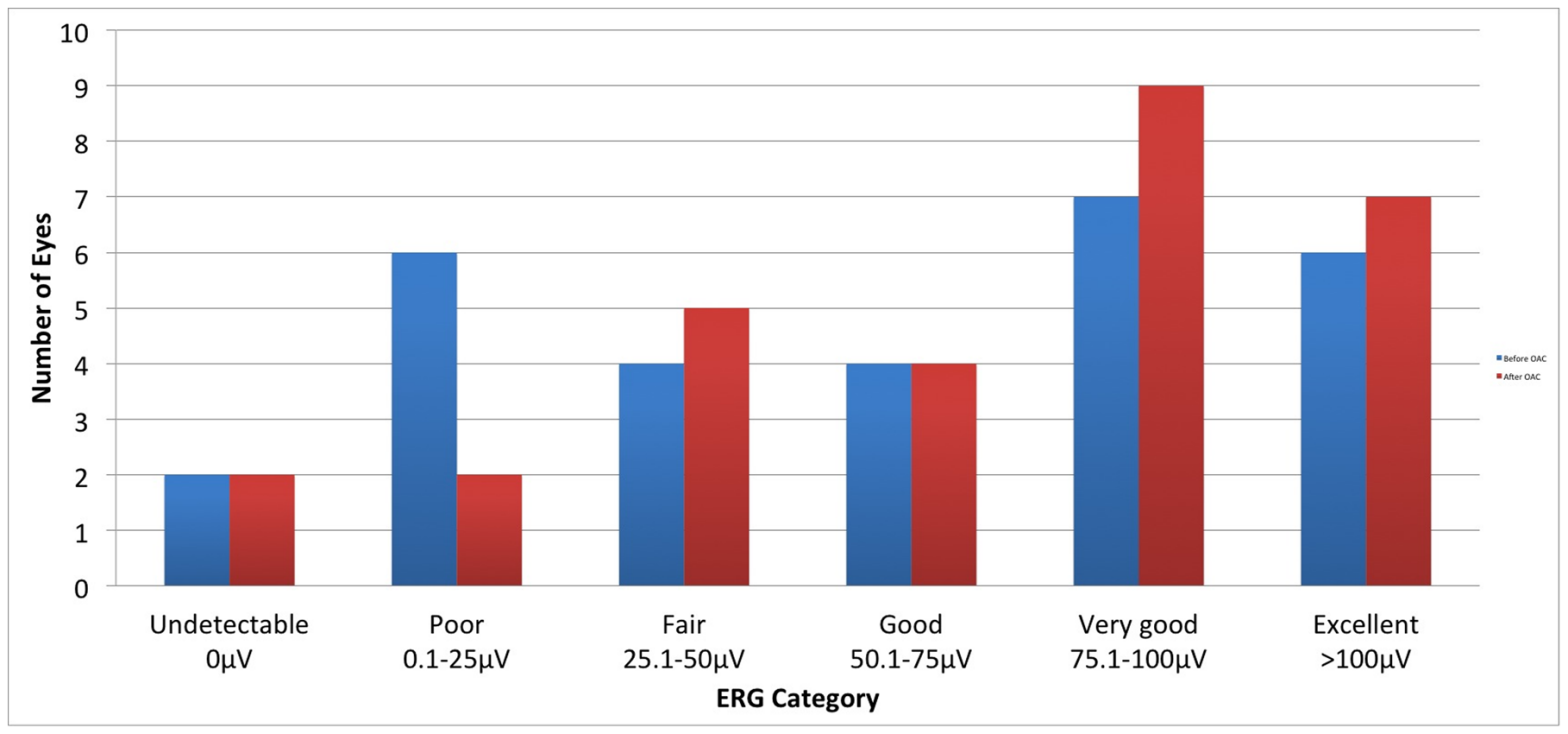
Electroretinogram categories before and after carboplatin +/− topotecan containing Ophthalmic Artery Chemosurgery. OAC = ophthalmic artery chemosurgery, ERG =  electroretinogram.

By univariate regression analysis, maximum carboplatin dose had no statistically significant negative effect on the ERG responses at three months (p = 0.59), nor at one year (p = 0.64) after OAC completion. By the same analysis, cumulative carboplatin dose had no statistically significant detrimental effect on ERG responses at three months (p = 0.96), nor at one year (p = 0.58). Likewise, neither maximum nor cumulative topotecan had any significant effect on ERG responses at 3 or 12 months. At last follow-up, the responses in eyes were classified into the ERG categories as shown in figure 3. In summary, ten eyes (10/29, 34%) had demonstrated an improvement of the responses by at least 25 µV (6 of which had resolution of previous retinal detachment following treatment), 16 eyes (16/29, 55%) were stable, and three eyes (3/29, 10%) developed a decrease in the ERG response of at least 25 µV (including two eyes with ERGs which were reduced from the “excellent” the “very good” range). The ERG responses before and after infusions of carboplatin-only (p = .07) or carboplatin with topotecan (p = 0.39) were not statistically significantly different.

### Hematologic Toxicity

There were 15 infusions in which melphalan was not used in either eye, and in which children were exposed to carboplatin and or carboplatin/topotecan during OAC to one or both eyes. Nine infusions were evaluable and of these, one patient developed Grade 3 neutropenia and Grade 2 anemia and one patient developed Grade 4. Both patients demonstrated this during their preceding OAC infusion when the melphalan dose equaled or exceeded 0.4 mg/Kg. Neither patient required a transfusion nor required filgrastim.

### Ocular Toxicity

Two eyes developed a temporary inflammatory syndrome with erythema and periorbital edema [17]. One patient, with plasminogen-activator inhibitor-1 polymorphism experienced an occlusive chorioretinopathy in both eyes following the second and last tandem OAC (when both eyes are treated during the same session), with recovery of useful vision (ERG response improving from poor to fair) [20]. Three patients developed transient periocular erythema in the distribution of the supra-trochlear artery along with medial ciliary madarosis – in all cases this was observed in conjunction with melphalan infusions. Two patients developed cataracts, one after the temporary inflammatory syndrome noted above and one patient in conjunction with a long-standing retinal detachment. One patient was found to have a temporary sixth nerve palsy 6 months following OAC completion in which two of four infusions were carboplatin based.

## Discussion

Since its introduction by French investigators nearly twenty years ago [6], carboplatin has been the most used drug used worldwide for intraocular retinoblastoma. More than 100 publications have supported its use [30]: carboplatin has been used as a single agent [31], in combination with one [32] or two additional drugs (vincristine and etoposide) [6], [7], [33] and in some cases with the addition of pulsed high dose Cyclosporine [34]. Expected side effects of systemically administered multi-agent chemotherapy that have been recorded include cytopenia, fever/neutropenia and, very uncommonly, death [35]. Recently there has been increasing concern as longer follow-up has demonstrated more ototoxicity than predicted [36]–[38]. Fatal, secondary leukemia has also been reported, though it is impossible to estimate the actual contribution of carboplatin, since those patients received multiagent chemotherapy and there is a greater risk of leukemia in patients receiving etoposide [39]. Based on children with other cancers, there is concern about future sterility or infertility [40] but the children who have received systemic carboplatin are only now approaching childbearing age and data is limited.

Intravenous carboplatin given as a single agent routinely causes a 30–50% reduction in tumor area and basal diameter within one month after just one dose of chemotherapy [31]. Unfortunately in clinical practice carboplatin is rarely (if ever) curative alone and ophthalmic oncologists have learned that as soon as the chemotherapy is stopped, regrowth develops within weeks to months. That is why additional treatments are needed to salvage the eye and arrest tumor growth despite as many as 6–9 cycles of carboplatin-containing chemotherapy. Although many eyes can be salvaged with a systemic carboplatin based regimen [9], eyes with vitreous seeding or subretinal seeding (which represent 75% of cases in the U.S.) fare less well [41], [42]. The situation is even worse for eyes with subretinal seeding as some recent series report success rates of 0% [43].

When our group began administering carboplatin by periocular injection, our non-human primate model demonstrated that in comparison with intravenous administration, the drug levels after periocular injection were seven to nine times higher in the eye and 90% less in the blood [44]. Though there were fewer effects on bone marrow, periocular injections cause (in 50% of cases) severe local reactions including scarring and, rarely, loss of vision [45], [46].

Notably, however, measurable tumor responses occurred in patients who had failed systemic carboplatin –that is, the same drug in higher concentrations was effective after failing with the lower dose [10]. This stimulated us to hypothesize that higher concentrations of drug, delivered via the ophthalmic artery, might be even more effective.

It was the Japanese investigators [12] who demonstrated that melphalan was highly effective in the laboratory and their recent report on 25-year follow-up [14] confirms the reproducible potency of intraarterial chemotherapy. The Japanese developed a tiny balloon catheter fed from the groin (femoral artery), expanded in the internal carotid artery to stop blood flow just above the origin of the ophthalmic artery, so that injected chemotherapy was all directed to the ophthalmic artery.

Nearly seven years ago we began cannulating the ophthalmic artery (from the femoral artery) and infusing melphalan directly into the orifice of the ophthalmic artery – a technique we term ophthalmic artery chemosurgery (OAC). We initially chose melphalan, building on the impressive experience of the Japanese. However, as we began treating both eyes with OAC during the same session (Tandem therapy) [47], the myelosuppressive effect of melphalan became dose limiting. Given the historical experience of using carboplatin to treat retinoblatoma, administering carboplatin via OAC was a logical next step. The direct infusion of drug into the ophthalmic artery has the advantage of delivering a high concentration of carboplatin to the eye, while minimizing the systemic exposure to the drug. Using non-tumor bearing experimental models as a guide for calculations, carboplatin dose increases seven fold when moving from intravenous to periocular applications [44]; and using a porcine model of intraarterial melphalan administration, vitreous drug levels likely increase again with OAC [48].

This report summarizes our experience with carboplatin +/− topotecan OAC, which proves to be an efficient delivery method for this drug with minimal local and systemic toxicity. There were no event-related deaths or adverse events related to anesthesia. While OAC benefits from dispensing a targeted drug with limited systemic exposure, it may result in myelosuppression in certain instances. With the drugs employed at the doses chosen, melphalan is expected to be the most myelosuppressive and carboplatin the least. To this point, OAC melphalan has resulted in significant neutropenia in some patients, predominantly in those with doses surpassing 0.4 mg/Kg [17]. In the present study, the mean maximum dose (average of single highest dose for each patient) of OAC carboplatin received by our patients was one sixth the dose they would have received systemically for tumor reduction (32 mg for OAC vs 192 mg for systemic administration, the calculated dose that would have been given for 18.7 mg/Kg) – giving us a low expectation for systemic toxicity. Furthermore, in phase I trials, the only significant systemic toxicity of topotecan is myelosuppression resulting in a dose-limiting level of 1.3 to 1.9 mg/m^2^/day (administered via 72-hr continuous infusion, with escalated cumulative dose per cycle) [49]. Our maximum topotecan dose ranges between 0.3 mg and 2.0 mg (mean of 0.5 mg and median of 0.4 mg) and is below these tolerable doses. In our patients exposed to carboplatin or carboplatin/topotecan during their single or tandem OAC, only two patients developed neutropenia.

Just like previous papers focusing on melphalan-based OAC had occasionally received additional drugs and treatment, almost all eyes included here received carboplatin +/− topotecan-containing OAC in conjunction with melphalan-containing OAC or with focal therapy. Eyes receiving carboplatin +/− topotecan-containing OAC had a measurable tumor response and the two eyes treated with carboplatin and laser demonstrated tumor control. Carboplatin +/− topotecan-containing OAC can allow for prompt regression of tumors and can be curative as a single agent in combination with focal techniques. Ophthalmic artery infusions of carboplatin +/− topotecan can be particularly useful in bilateral patients receiving tandem therapy where the risk of myelosuppression with melphalan may be dose limiting [19].

Overall, eyes with carboplatin +/− topotecan-containing OAC as included in this study, demonstrated ocular survival of 89.9% at two years. Because we have previously reported on the use of triple-agent OAC [24], including carboplatin, these patients were not included in this series. Had they been included, the ocular survival for any patient who received carboplatin would probably be less. Since prior treatment typically involves systemic carboplatin, it may come as a surprise that eyes receiving this same drug via an intraarterial route would fare well; perhaps the efficacy lies in the higher, localized dose or the addition of other drugs. These findings suggest that ocular survival at two years is no worse for carboplatin +/− topotecan-containing OAC compared to melphalan-predominant OAC, and is effective in eyes that have received previous systemic carboplatin.

While an elevated dose may be adequately effective, it also raises concern for increased toxicity. Animal studies suggest a dose-dependent impact of carboplatin on the choroid and retina [50]. This dose dependent toxicity has been further corroborated by ERG findings in animal models [51]–[53] with an inner retina effect measurable by ERG. In humans, retinal toxicity was “dose-limiting” for intracarotid administration of carboplatin (mean 300 mg/m2) for treatment of brain tumors [54]. Even though these doses are ten-fold higher than those employed in carboplatin OAC, they still demonstrate the potential for chorioretinal toxicity with carboplatin. As such, we used electroretinogram measurements to monitor for retinal toxicity.

Carboplatin OA therapy had no significant effect on ERG responses at three months, nor one year when evaluated by maximum carboplatin or cumulative carboplatin dose. In addition, analysis of ERG responses following infusions containing carboplatin only and carboplatin with topotecan revealed no statistically significant change. Furthermore 90% of eyes had stable or improved responses when comparing electroretinograms before and after treatment. This suggests that at the doses being employed, intraarterial carboplatin, or topotecan, has no significantly measurable toxicity to the retina.

Carboplatin is a well-established drug in the treatment of retinoblastoma, which has now proven useful via the relatively novel route of OAC. Carboplatin has several advantages which distinguishes it from melphalan: it causes less systemic toxicity, is not associated with medial periocular erythema, and is easier to infuse for two reasons: it requires no filtration prior to administration and its stability deems it time insensitive, meaning it can be injected even in cases of prolonged ophthalmic artery cannulation (beyond 30 mins). This work represents a comprehensive report on carboplatin +/− topotecan-containing OAC for retinoblastoma, demonstrates encouraging results and supports its use as an efficacious addition to melphalan, or in some selected cases, an alternative to melphalan with both a limited ocular and systemic toxicity profile. Its superiority to melphalan OAC is yet to be determined.
